# Nestedness and beta diversity of gastrointestinal helminth communities in common warthogs, *Phacochoerus africanus* (Suidae), at 2 localities in South Africa

**DOI:** 10.1017/S0031182023000719

**Published:** 2023-09

**Authors:** Kerstin Junker, Ivan G. Horak, Joop Boomker, Boris R. Krasnov

**Affiliations:** 1 National Collection of Animal Helminths, Epidemiology, Parasites and Vectors Programme, ARC-Onderstepoort Veterinary Institute, Private Bag X05, Onderstepoort 0110, South Africa; 2 Department of Veterinary Tropical Diseases, University of Pretoria, Private Bag X04, Onderstepoort 0110, South Africa; 3 Mitrani Department of Desert Ecology, Swiss Institute for Dryland Environmental and Energy Research, Jacob Blaustein Institutes for Desert Research, Ben-Gurion University of the Negev, Sede Boqer Campus, 8499000 Midreshet Ben-Gurion, Israel

**Keywords:** Beta diversity, community structure, dark diversity, helminths, nestedness, *Phacochoerus africanus*, South Africa

## Abstract

Few studies have investigated the ecological interactions between wild species of Suidae and their parasites, leaving our knowledge concerning this host–parasite system fragmented. In the present study, we applied network studies to analyse community nestedness in helminth assemblages of common warthogs, *Phacochoerus africanus* (Gmelin) (Suidae). Helminth data were compiled from 95 warthogs, including young and adult males and females, from 2 different conservation areas in Mpumalanga and Limpopo Provinces, South Africa, collected monthly over a period of 1 year each. The aim was to study the effect of host sex, age and season of sampling on the structure of helminth infracommunities harboured by the warthogs and to search for non-random structural patterns in the warthog–helminth interaction networks. Furthermore, we investigated the influence of a warthog's age, sex and season of sampling on beta diversity and dark diversity of their helminth infracommunities. Lastly, we asked whether the effects of host sex, age and sampling season on helminth communities differed between the 2 localities. We found that helminth communities of warthogs were nested and host–parasite interactions were influenced by all 3 factors as well as combinations thereof. However, the resulting patterns differed at the 2 localities, indicating that local environmental processes are important drivers of community structure.

## Graphical abstract

**Figure:**
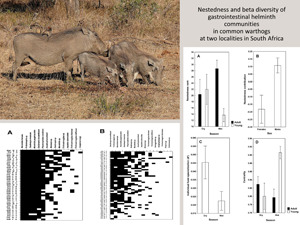

## Introduction

One of the key aims of studies of host–parasite interactions is understanding host characteristics and environmental factors that influence parasite transmission dynamics. The effects of host sex, age and season of sampling on parasite abundance, prevalence and species richness have been studied repeatedly, often with varying outcomes depending on the host or parasite taxa studied or the spatial or temporal context (Isomursu *et al*., 2006; Saeed *et al*., 2006; Martínez-Guijosa *et al*., 2015; Amundson *et al*., 2016; Spickett *et al*., 2017).

In recent years, parasite ecologists focused their efforts on the effects of these factors on parasite community structure. Studies aiming to elucidate if parasites live in structured communities or in unstructured assemblages were conducted mainly with a view to parasite communities in fish hosts (Guégan and Hugueny, 1994; Rohde *et al*., 1998; Carney and Dick, 2000; Norton *et al*., 2004), amphibians (Zelmer *et al*., 2004; Martins *et al*., 2021), birds (Calvete *et al*., 2003; Amundson *et al*., 2016) and small mammals (Warburton *et al*., 2018; Krasnov *et al*., 2019; Rynkiewicz *et al*., 2019; Cardoso *et al*., 2021).

However, relatively few studies dealt with parasite communities of large mammalian hosts. This is because it is often difficult to obtain multiple samples of large mammals, since population numbers have shrunk as a consequence of habitat loss due to human activities, and populations are limited to relatively small conservation areas. In addition, the sheer effort of processing large hosts for parasite sampling and identification often proves prohibiting. Nevertheless, 1 such study on a large marine mammal species was conducted by Bellay *et al*. (2020). These authors investigated the influence of host age and sex on infracommunities in a pilot whale–helminth network and concluded that host age was one of the main drivers of the occurrence and species richness of intestinal helminths. Moreover, they found that the increase in parasite richness within a host resulted mainly from non-random infection with helminths.

Recently, we studied the impact of host sex and age on the structure of individual-based host–parasite networks in nyalas, *Tragelaphus angasii* Angas, from 3 game reserves in South Africa (Junker *et al*., 2022). We found that the effects of both host sex and age can be mediated by local environmental conditions and may manifest differently in the same host but sampled in different localities. Similar work on wild ruminants was done by Fellis *et al*. (2003) and Negovetich *et al*. (2006) in southern Africa. Fellis *et al*. (2003) found non-randomness in parasite communities of greater kudu, *Tragelaphus strepsiceros* (Pallas), in the Kruger National Park (KNP) and, comparing these to parasite communities of kudus in Etosha National Park, Namibia, concluded that biogeography and host demographics played an important role in the organization of helminth communities. Negovetich *et al*. (2006) demonstrated the influence of social and feeding behaviour of different age-sex classes of impalas, *Aepyceros melampus* (Lichtenstein), on parasite communities. These findings may have important consequences for planning and designing parasitological investigations. However, it is still unclear whether environmental mediation of the effects of host-associated factors on parasite community structure found for parasite infracommunities in nyalas, kudus and impalas also holds for other host species.

Comprehensive data sets on the gastrointestinal parasites of common warthogs, *Phacochoerus africanus* (Gmelin) (Suidae), compiled by Horak *et al*. (1988) and Boomker *et al*. (1991), allowed us to address this gap. Monogastric warthogs differ both in their sociality and dietary preferences (see below) from the above ruminant species, which are more gregarious and intermediate mixed feeders or browsers (Skinner and Chimimba, 2005). To date, few studies have investigated helminth communities in wild species of suids (Corn *et al*., 1985; de-la-Muela *et al*., 2001; Foata *et al*., 2006). Junker *et al*. (2019), having studied the influence of host age and sex on infrapopulations of gastrointestinal helminths of warthogs in the KwaZulu-Natal Province in South Africa, emphasized a need to further explore helminth assemblages and their structure in wild suids in Africa.

Here we studied the effects of host sex, age and season of sampling on the structure of helminth infracommunities harboured by warthogs at 2 localities in South Africa. First and similar to our earlier study on the parasites of nyalas, we searched for non-random structural patterns in the warthog–helminth interaction networks. Among a variety of ecological structures, nestedness (i.e. a pattern where species found in species-poor communities represent a proper subset of species in richer communities) is one of the most common properties of species distributions (Guégan and Hugueny, 1994; Poulin and Valtonen, 2001; Krasnov *et al*., 2010; Rynkiewicz *et al*., 2019). Then, we investigated contributions of male and female young and adult warthogs sampled during dry and wet seasons to this nested pattern. As mentioned above, similar work had been done by Fellis *et al*. (2003). However, Fellis *et al*. (2003) used the matrix temperature as a measure of nestedness (Patterson and Atmar, 1986; Atmar and Patterson, 1993). This methodology has been subject to severe criticism because this metric is susceptible to type 1 errors and, especially in communities comprising mainly ubiquitous and rare species, the use of this metric might lead to false conclusions (Ulrich *et al*., 2009). Here, we applied the most popular nestedness metric, the Nestedness based on Overlap and Decreasing Fill (NODF) index (Almeida-Neto *et al*., 2008; Ulrich *et al*., 2009). This index is robust to changes in matrix shape or size and can vary from zero in a not nested (=random) network to 100 in a perfectly nested network. One of the advantages of the NODF index is that it quantifies nestedness for not only the whole matrix but also nestedness independently contributed by matrix rows (in our case, nestedness among helminth species) and matrix columns (in our case, nestedness among host individuals).

Secondly, we considered beta diversity of helminth infracommunities harboured by warthogs (i.e. helminth diversity between individual warthogs). Legendre and De Cáceres (2013) argued that beta diversity of a set of communities is affected by both individual species and individual species assemblages (=sites) and developed a method that allows to separate these effects. In other words, total beta diversity can be partitioned into (a) the degree of relative importance of individual species for beta diversity across an area (=Species Contributions to Beta Diversity; SCBD) and (b) indicators of the compositional uniqueness of the assemblages as compared to other sites (=Local Contributions to Beta Diversity; LCBD). This partitioning allows a better understanding of the differential roles of species and assemblages in total beta diversity. Here, we focused on LCBD, which in application to parasite communities should be called Host Contributions to Beta Diversity (HCBD), and tested for the associations between warthog sex, age and season of sampling and HCBD to elucidate the reasons behind the compositional uniqueness of helminth infracommunities.

Thirdly, we asked whether warthog sex, age and season of sampling affected dark diversity of their helminth infracommunities. In contrast to realized diversity (i.e. species that are present in a locality), dark diversity (Pärtel *et al*., 2011) is defined as the assemblage of species from the regional pool that could inhabit this locality due to suitable conditions but that are, in reality, absent. In application to parasite infracommunities, an individual host of a given species is analogous to a locality, whereas parasite species harboured by all hosts of this species in the same geographic region or habitat type are analogous to a regional species pool because they are able to exploit this host species. Estimations of parasite dark diversity allow to identify the gaps between parasite species richness in an individual host and a parasite species pool and comparisons of these gaps (i.e. dark diversity estimates) between individual hosts may allow better predictions and preventions of parasitic disease outbreaks. Estimation of dark diversity requires definition of a species pool, which cannot be arbitrarily established from species inventories because this approach (a) ignores species’ ecological constraints and the interspecific variation in these constraints (Lewis *et al*., 2016) and (b) inventories, obviously, cannot account for species that are a part of dark diversity.

Thus, as presented above, we sought to uncover the effect of host sex, age and season of sampling on: (1) patterns of nestedness in warthog–helminth networks, (2) the contribution of species assemblages in individual warthogs (HCBD) to total beta diversity, and (3) dark diversity of helminth infracommunities of warthogs.

Finally, we asked whether the effects of host sex, age and sampling season on structural patterns differ between the 2 localities because of local environmental processes. Altogether, answering these questions will allow us to determine internal and external factors that serve as drivers of the community structure of helminths of warthogs.

## Materials and methods

### Host species

Common warthogs are widespread in sub-Saharan Africa. With exception of the forest zone, their range extends westwards from Senegal to Ethiopia and southwards to South Africa. Within South Africa, warthogs are common in the Limpopo and Mpumalanga Provinces and in the north-eastern parts of North West Province. Their preferred habitats are savanna, grassland, open woodland and bushland, but they also frequent floodplains and wetlands. Warthogs are diurnal and spend the day foraging for grasses, especially those that are fresh and green, herbs, berries and wild fruits. They will also root for underground rhizomes of grasses. Their diet is seasonally variable with more time spent rooting during winter (June to September), whereas grazing is preferred in summer. Males (59.2–103.9 kg) are larger than females (44.6–69.1 kg) (Skinner and Chimimba, 2005). The size of warthog groups is usually small. Adult males are typically solitary, but mingle with other warthogs at waterholes, while younger males of more than 1 year of age join bachelor groups of 2–3 individuals. Matriarchal groups comprise 1 or more females with their juvenile and sometimes yearling offspring, although yearlings eventually move off to form yearling groups that exclude any other age classes (Somers *et al*., 1995). Warthogs are not territorial and groups occupy overlapping home ranges. The mating system is promiscuous, with males moving between the home ranges of several females; females will mate with more than 1 male (Somers *et al*., 1994; Skinner and Chimimba, 2005).

### Study areas

Warthogs were collected from 3 sampling sites within the KNP in Mpumalanga Province (see Horak *et al*., 1988) and from 1 sampling site in the Hoedspruit Nature Reserve (HNR; now AFB Game Reserve; 24°21’17”S, 31°03’01”) in Limpopo Province, South Africa (see Boomker *et al*., 1991). The sites in the KNP were the areas around the Skukuza (24°59’43”S, 31°35’34”E), Crocodile Bridge (25°21’30”S, 31°35’32”E) and Lower Sabie (25°7’16”S, 31°55’2”E) rest camps. All collection sites are situated in the Savanna Biome in the Lowveld bioregion in a vegetation zone classified as Granite Lowveld (Mucina and Rutherford, 2006). The climate is characterized by hot summers and mild, usually frost-free winters. Winters are dry, with the main rainfall occurring in summer.

Climate data were recorded during the sampling period at Skukuza (January 1980 to January 1981) and in the HNR (August 1988 to July 1989). At Skukuza, a minimum temperature of 3°C was recorded in June 1980 and a maximum of 33°C was reached in January 1981. A total of 748 mm of rain (0–160 mm) was measured in the 13-month period (see Horak *et al*., 1988). At HNR, the minimum temperature recorded in July 1989 was 10.4°C, the maximum was 30.7°C in March 1989, with a total of 350 mm of rain (0–108 mm) in the twelve-month period (see Boomker *et al*., 1991).

At the time of sample collection, the HNR comprised approximately 4000 ha, owned by the South African Defense Force. Within this reserve, and covering a restricted area of about 2000 ha, the Air Force Base Hoedspruit was situated. Warthogs were collected in both the open and restricted area of the reserve. A series of security fences between the 2 areas prevented warthogs from either side of the fence from passing through. In contrast, the outer fencing between the HNR and adjacent privately owned game farms, did allow warthogs to pass freely.

### Host and parasite collection

A total of 54 warthogs (23 females, 31 males) were collected on a monthly basis from January 1980 to January 1981 in the KNP. Of these, 10 were adult females, 13 young females, 18 adult males and 13 young males. Nineteen warthogs were collected in the dry season (May to August), whereas 35 were collected in the wet season (September to April). With the exception of March 1989, when none could be located, warthogs were collected on a monthly basis from August 1988 to July 1989 at HNR. In total, 41 warthogs (25 females, 16 males) were collected, including 14 adult females, 11 young females, 6 adult males and 10 young males. Of these, 16 were collected during the dry season, 25 in the wet season. Warthogs from 1 to 24 months of age were classified as young animals, and animals >24 months of age as adults.

Warthogs were processed for worm recovery as detailed in Horak *et al*. (1988) and Boomker *et al*. (1991). Briefly, carcasses were eviscerated and each gastrointestinal tract divided into stomach, small intestine and large intestine. The contents of these sections were removed and aliquots made from the ingesta. In addition, digests and aliquots thereof for the recovery of fourth-stage larvae (*Impalaia tuberculata* L4 and *Trichostrongylus* sp. L4) were made of the mucosae of the GIT sections of warthogs in the KNP, but not for warthogs in the HNR. Parasites were collected from the aliquots, identified and counted. Aliquot counts were subsequently converted into full counts. *Schistosoma* sp. was collected from the mesenteric veins. Parasites were identified using relevant keys and taxonomic works. Species and their authorities are listed in Supplementary Tables 1 & 2.

As it is often difficult to distinguish congeneric females within the Strongylida, we have assigned females in a host individual to the various species based on the ratio of males. If males were absent, females could only be identified to genus level. To date, 2 species of the cestode genus *Moniezia* Blanchard, 1891 have been described from warthogs, *Moniezia mettami* Baylis, 1934 and *Moniezia phacochoeri* (Baylis, 1927) (syn. *Paramoniezia phacochoeri* Baylis, 1927) (Beveridge, 2014). Because of time constraints, specimens were only identified to genus level and are treated here as *Moniezia* sp. Counts concerning *Moniezia* sp. were based on scoleces. Representatives of the nematode genus *Probstmayria* Ransom, 1917 recovered from warthogs in South Africa have generally been assigned to *Probstmayria vivipara* (Horak *et al*., 1988; Boomker *et al*., 1991; Junker *et al*., 2019). However, during the course of this study, we found the description of *Probstmayria suis* Troncy, Graber and Thal, 1927 as a new species from *P. africanus* (as *Phacochoerus aethiopicus* (Pallas)) and *Hylochoerus meinertzhageni* Thomas in the Central African Republic (Troncy *et al*., 1972). According to the latter authors, differentiation between the females of the 2 species in the absence of males, which are extremely scarce, is not possible. Since no males could be examined, we record the specimens here as *Probstmayria* sp. Counts of *Probstmayria* sp. were in their millions and are therefore not further specified.

### Data analysis

An interaction warthog–helminth network for each of the 2 localities was represented by a presence/absence matrix with individual warthogs in columns and helminths in rows. As mentioned above, we tested whether helminth infracommunities of warthogs demonstrated a nested pattern by the NODF index (Almeida-Neto *et al*., 2008; Ulrich *et al*., 2009). In the context of this study, we focused on nestedness among individual warthogs (i.e. nestedness among matrix columns). We calculated NODF using the program NODF 2.0 (Almeida-Neto and Ulrich, 2011) and determined its significance using a null model with fixed column (hosts) and equiprobable row constraints (Gotelli, 2000) with 1000 permutations. This algorithm does not constrain the number of helminth species that can occur in a host individual (see Krasnov *et al*., 2006 for justification of this model in application to host–parasite associations). Prior to calculations of NODF, each matrix was sorted according to helminth species richness (i.e. maximally packed matrix). Then, following Wang *et al*. (2013) and Chen *et al*. (2022), we derived the nestedness rank of each individual warthog in the maximally packed matrix from the default output.txt of the NODF program. In addition, for each individual warthog, we calculated Z-transformed nestedness resultant (Nres) and the nestedness contribution (Ncont) (Almeida-Neto and Ulrich, 2011). The Nres of an individual warthog is the standard effect size of the NODF metric where the observed degree of nestedness is compared to the degree after randomizing the occurrences of this warthog. The Ncont of an individual warthog represents the difference between the NODF metric calculated when this individual was excluded and the NODF metric calculated when it was included. Therefore, it indicates whether the matrix nestedness increases (Ncont < 0) or decreases (Ncont > 0) after this individual is excluded from the calculations.

Then and following Junker *et al*. (2022), we estimated the importance of each individual warthog in a network using 3 indices. The index of individual host (=warthog) specialization (*d’*) represents the deviation from a neutral configuration of associations (Blüthgen *et al*., 2006; Blüthgen, 2010) by comparison of the frequency distribution of interactions with a null distribution when interactions between warthogs and helminths are proportional to their observed total frequencies. This index may vary from 0 (no specialization) to 1 (complete specialization). Individual host strength (IHS) is a species-strength index of Bascompte *et al*. (2003) calculated as the sum of the dependencies of each helminth species on each individual warthog. d’ and IHS were calculated using function ‘specieslevel’ of the R package ‘bipartite’ (Dormann *et al*., 2008; Dormann, 2011). Centrality (C) assesses the role of each warthog in sharing helminths with other warthogs within a locality. High centrality of a warthog would indicate its greater connection with other warthogs thus would suggest high level of helminth transmission (Morand *et al*., 2014; Pilosof *et al*., 2015). To calculate centrality, a bipartite network was projected to a unipartite network, which was subsequently transformed into the network graph object. This was done using the R package ‘tnet’ (Opsahl, 2009). C was then calculated as eigenvector centrality using the R package ‘igraph’ (Csardi and Nepusz, 2006).

To calculate host contribution to beta diversity (HCBD) of helminth infracommunities, we first transposed each network matrix, so that columns represented helminth species, whereas rows represented individual warthogs. Subsequently, we calculated HCBD values for each individual warthog using function ‘beta.div’ and Hellinger dissimilarity coefficient implemented in the R package ‘adespatial’ (Dray *et al*., 2018). In addition, we partitioned total beta diversity of helminth infracommunities into spatial (=between-host) turnover and nestedness components (Baselga, 2010) for the sake of comparison of nestedness component of infracommunity beta diversity between the 2 areas (HNR and KNP). This was done using the function ‘beta.multi’ of the R package ‘betapart” (Baselga *et al*., 2023).

Estimation of dark diversity can be done *via* calculation of the probability of species occurrence based on the co-occurrence of a given species with other species. In this approach, a species that is absent from a given site is considered to be part of the species pool if it usually co-occurs with species present in this site (Ronk *et al*., 2015; Lewis *et al*., 2016). For each species, the co-occurrence probability is calculated in the sites where it is actually present and the sites where it is absent. Subsequently, a species is included in dark diversity if it is absent in a site, but its occurrence probability is greater than some threshold (Ronk *et al*., 2015). Carmona and Pärtel (2021) presented a method to estimate probabilistic species pools using pairwise co-occurrence data in which species suitability in a site is directly estimated by comparing the realized co-occurrence pattern of each species pair to that expected if there is no association between these species, while the extent of the departure of the observed co-occurrence between the species pair from random association is used as the indicator value for this pair. The best method to calculate the probability of co-occurrence proposed by Carmona and Pärtel (2021) involved the calculation of the expected number of co-occurrences as the mean value of the hypergeometric distribution (Arita, 2016). Here, we used the hypergeometric method and calculated the suitability (=probability to belong or not belong to dark diversity) of each helminth species absent from an individual warthog. The probability of an absent helminth to belong to the dark diversity of an individual warthog is high if its associations with the helminths present in other warthogs are mostly positive, whereas the opposite is true if its associations with the present helminths are negative. We calculated the probabilities of helminths to belong to the dark diversity using the R package ‘DarkDiv’ with the options ‘method = hypergeometric’ and ‘wa = F’ (because numbers of helminths belonging to different species cannot be compared). The helminth dark diversity size for each individual warthog was calculated as the sum of the probabilities of all helminth species absent from this warthog to belong to dark diversity.

To test for the associations between a warthog's sex, age and season of sampling (explanatory variables) and each of the nestedness metrics as well as dark diversity size (response variables), we used generalized linear models (GLMs). In GDMs of d’ and C, the option ‘family = quasibinomial’ was applied because these indices may vary between 0 and 1 and may attain either 0 or 1. In contrast, we applied beta-regression (Cribari-Neto and Zeileis, 2010) with a logit link function for the analyses involving HCBD because the HCBD value ranges from 0 to 1 without attaining either 0 or 1. Beta-regression approach is the most suitable for models with a response variable of this type because this approach incorporates heteroscedasticity and skewness characteristics for these variables. Beta-regressions were carried out using the R package ‘betareg’ (Cribari-Neto and Zeileis, 2010). For each metric, we ran a number of models with all possible combinations of the explanatory variables, including an intercept-only model, and then selected the best model based on Akaike information criterion using the R package ‘MuMIn’ (Barton, 2020).

## Results

Warthogs collected at the 2 localities in South Africa harboured a total of 18 gastrointestinal helminths, comprising 16 species of nematodes, 1 species of cestode and 1 species of trematode (Supplementary Tables S1 and S2). Of these, 17 species were present at KNP, with only *Cooperia hungi* being absent, whereas 13 species were present at HNR. All 5 species absent from warthogs at HNR (*Strongyloides* sp., *Streptopharagus* sp., *Trichostrongylus falculatus*, *Trichuris* sp. and *Schistosoma* sp.), were scarce species at KNP, where they had a prevalence of less than 10% (Supplementary Table S1). Similarly, *C. hungi* infected a single adult female at HNR. By far the most prevalent species was *Probstmayria* sp., with a prevalence of 100% at both localities and being the only species present in an adult male at KNP. All warthogs were infected with at least 1 helminth species. While being hosts to a larger pool of species, species richness in warthogs at KNP was lower, with a mean of 5.3 ± 0.2 and a range of 1–9 species per host individual, whereas warthogs at HNR were infected with 5–10 species, with a mean species richness of 7.0 ± 0.2. With the exception of the indirectly transmitted nematodes *Physocephalus sexalatus* and *Streptopharagus* sp., the cestode *Moniezia* sp. and the trematode *Schistosoma* sp., helminths recovered from warthogs at the 2 localities have a direct life cycle (Supplementary Table S2).

Adults of *Murshidia hamata* and *Murshidia pugnicaudata* were amongst the most prevalent and abundant species at both localities, with *M. hamata* being the dominant of the 2 species, reaching a slightly higher prevalence and abundance than its congener (Supplementary Table S1). Equally prevalent and even more abundant at HNR than *Murshidia* spp. were adults of *Daubneyia mocambiquei* and *Daubneyia mwanzae*. Prevalence of these 4 species ranged between 48–83% at KNP and between 95–100% at HNR. Another common species at HNR with a prevalence of 81% was *P. sexalatus*, adults of which infected 37% of hosts at KNP (Supplementary Table S1).

Helminths with an intermediate prevalence, i.e. adults infecting less than 50% but more than 10% of hosts at both localities, were *Ascaris phacochoeri*, *I. tuberculata*, *Trichostrongylus thomasi* and *Moniezia* sp. The remaining species (*Strongyloides* sp., *Streptopharagus* sp., *C. hungi*, *Trichostrongylus deflexus*, *T. falculatus*, *Trichuris* sp. and *Schistosoma* sp.) had a prevalence of less than 10% (Supplementary Table S1).

**Figure 1.**
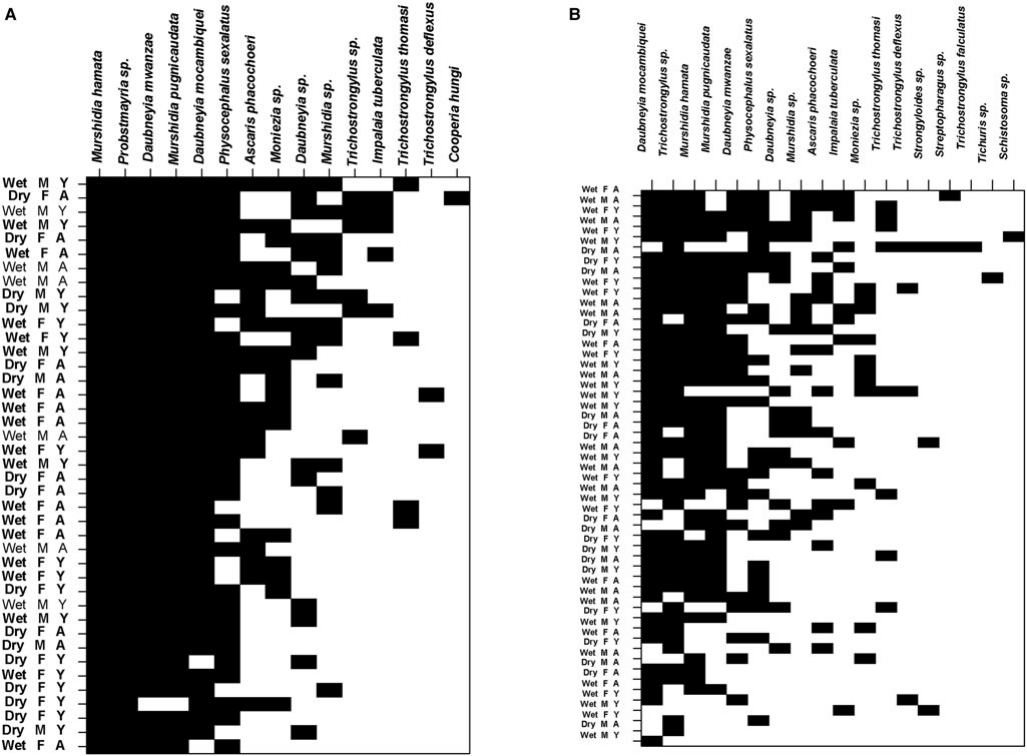
Maximally nested presence–absence matrices of individual warthog–helminth associations in Hoedspruit Nature Reserve (A) and Kruger National Park (B). M and F – male and female warthogs, respectively; A and Y – adult and young warthogs, respectively; Dry and Wet – warthogs sampled during dry and wet season, respectively.

**Figure 2.**
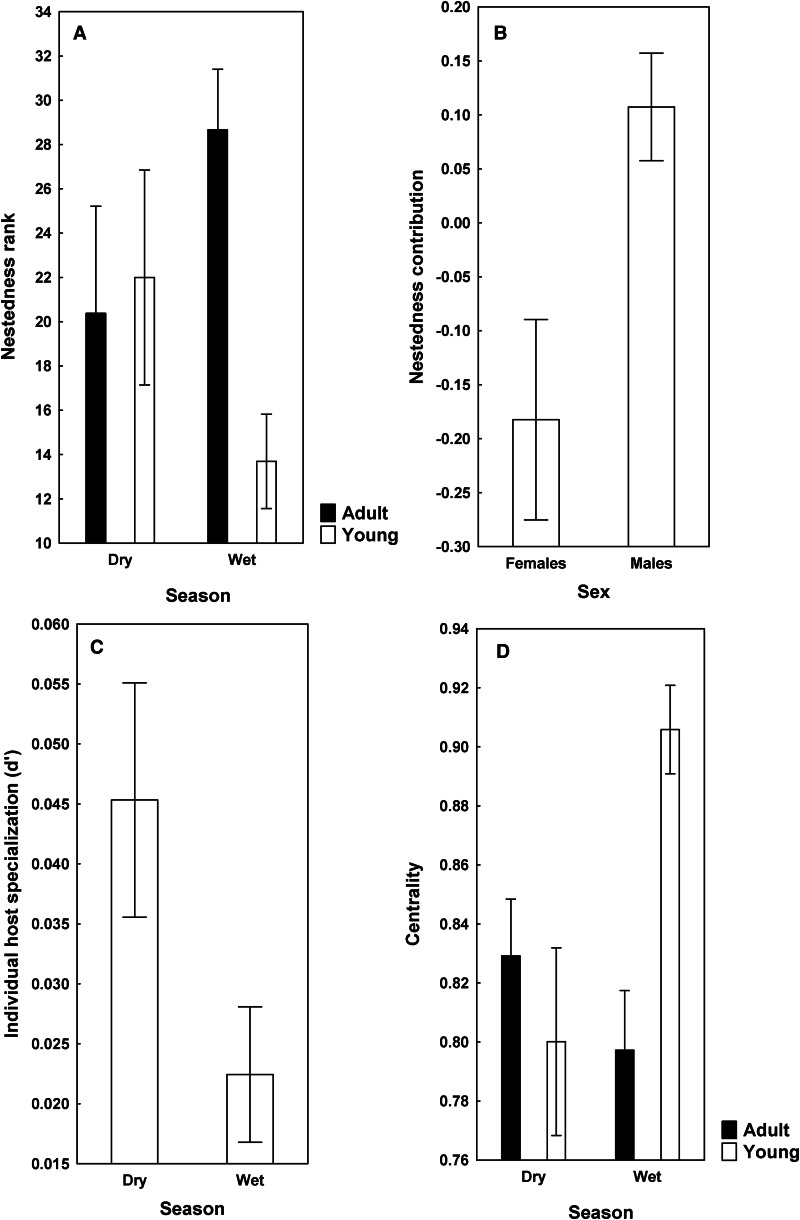
Mean (±s.e.) values of nestedness rank (A), nestedness contribution (B), individual host specialization (*d’*) (C), and centrality (C) in adult and young male and female warthogs sampled during dry and wet seasons from Hoedspruit Nature Reserve.

**Figure 3.**
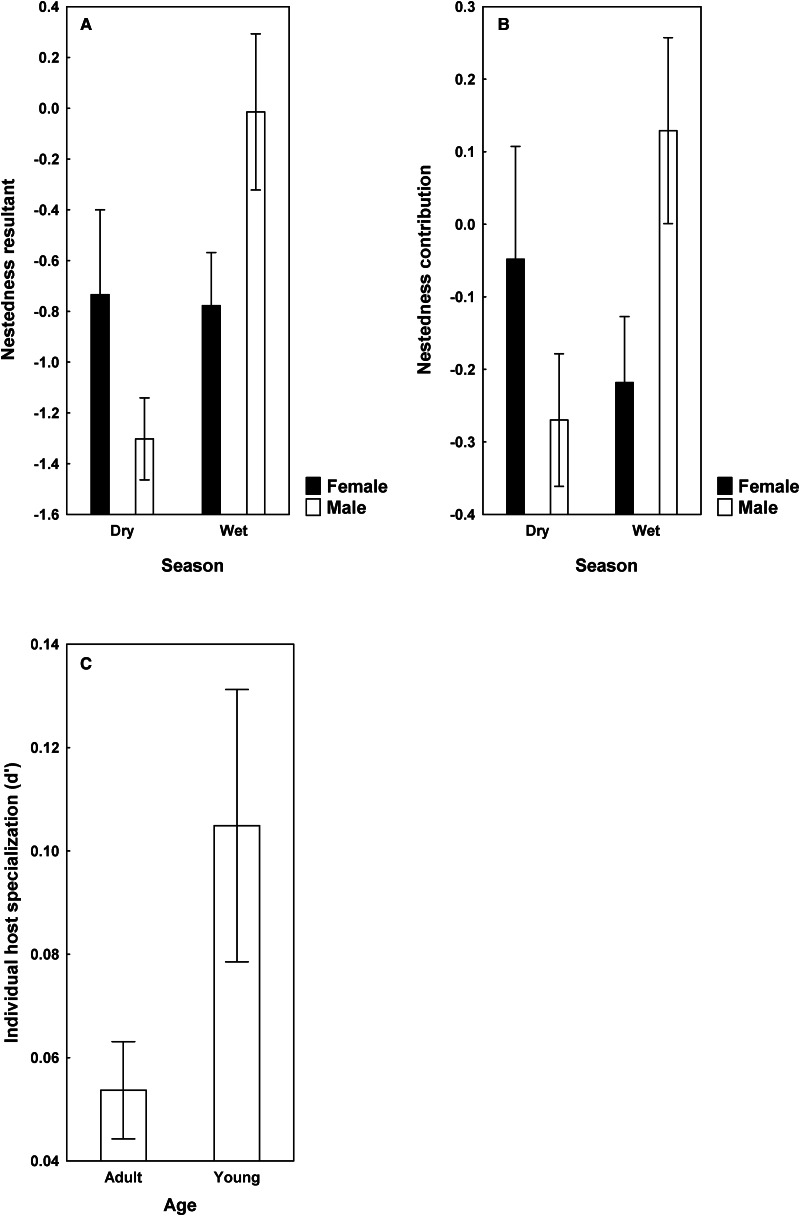
Mean (±s.e.) values of nestedness resultant (A), nestedness contribution (B), and individual host specialization (*d’*) (C) in adult and young male and female warthogs sampled during dry and wet seasons from Kruger National Park.

Warthog–helminth networks at the 2 localities are visualized on Fig. 1. Both networks were significantly nested, albeit the degree of nestedness in helminth infracommunities of warthogs from KNP was not especially high (NODFhosts = 73.43, Z = 55.57 for HNR and NODFhosts = 58.90, Z = 50.16 for KNP; *P* < 0.001 for both). This was further supported by partitioning of beta diversity of helminth infracommunities into species turnover and nestedness components with the nestedness component for warthogs in KNP being substantially lower than that for warthogs in HNR (0.04 *vs* 0.12, respectively). Summaries of GLM for the effects of a warthog's sex and age as well as season of sampling on nestedness metrics are presented in Table 1. Nestedness rank was higher in adult than in young warthogs, but this was only the case for animals sampled in the wet season in HNR (Fig. 2A). No effect of any explanatory variable on nestedness rank was found in KNP. Nestedness contribution was higher in male than female warthogs (a) independently of their age and season of sampling in HNR (Fig. 2B) and (b) in the wet season in KNP (Fig. 3B), whereas the opposite effect of sex on nestedness contribution was found in KNP in the dry season (Fig. 3B). In HNR, nestedness resultant did not respond to either explanatory variable, whereas in KNP, it demonstrated the same pattern as nestedness contribution in this area, being higher in females in the dry season and higher in males in the wet season (Fig. 3A, B). Individual host specialization (d’) was affected by season of sampling in HNR (higher in the dry season) (Fig. 2C) but by warthog age in KNP (higher in young individuals) (Fig. 3C). Values of centrality were higher in young than adult warthogs sampled in the wet but not the dry season in HNR (Fig. 2D), but no effect of host sex, age or season of sampling on this metric was found in KNP. The latter was also true for IHS in both localities.

**Table 1.** Coefficients of the best models of the effects of a warthog's sex (SX), age (A), season of sampling (SE) and their interactions on (a) the metrics of nestedness [nestedness rank (NR), nestedness resultant (Nres) and nestedness contribution (Ncont)], (b) indices of the importance of individual warthogs in a warthog–helminth network [individual host specialization (d’), individual host strength (IHS) and centrality (Cent)]; (c) contribution of each individual warthog to beta diversity of helminths in helminth infracommunities (HCBD); and (d) dark diversity size (DDS) of helminths in an individual warthog in Hoedspruit Nature Reserve (HNR) and Kruger National Park (KNP). Reference levels of explanatory variables were female for sex, adult for age and dry season for season. Only significant coefficients are shown. NR, Ncont, d’, Cent and DDS were modelled using generalized linear models (GLM), whereas HCBD was modelled using beta-regression.

Locality	Response variable	Explanatory variable	Coefficient ± s.e.	*t*	*P*
HNR	NR	A (young) × SE (wet)	−16.60 ± 6.92	−2.40	0.02
	Ncont	SX (males)	0.29 ± 0.12	2.35	0.02
	d’	SE (wet)	−0.72 ± 0.34	−2.12	0.04
	Cent	A (young) × SE (wet)	1.09 ± 0.32	3.38	0.001
	HCBD	SE (wet)	−0.27 ± 0.66	−2.35	0.02
	DDS	A (young)	−0.57 ± 0.22	−2.54	0.01
KNP	Nres	SX (males) × SE (wet)	1.33 ± 0.62	2.15	0.04
	Ncont	SX (males) × SE (wet)	0.57 ± 0.27	2.12	0.04
	d’	A (young)	0.72 ± 0.36	2.01	0.05

**Figure 4.**
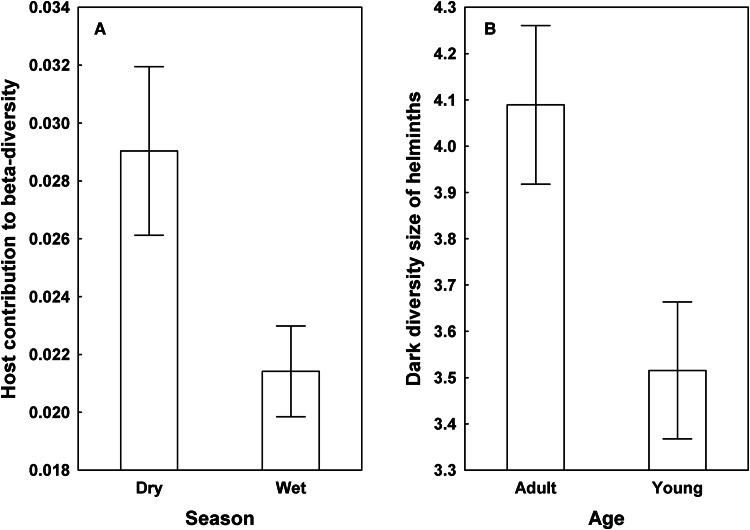
Mean (±s.e.) values of host contribution to beta diversity of helminth infracommunities (A) and dark diversity size of these communities (B) in adult and young warthogs sampled during dry and wet seasons from Hoedspruit Nature Reserve.

Effects of host-associated variables on community metrics unrelated to nestedness patterns (HCBD and dark diversity size) were revealed in HNR, but not KNP. In particular, contribution to beta diversity of helminth infracommunities differed between warthogs sampled in different seasons, being higher in the dry season in both adult and young female and male warthogs (Fig. 4A). Dark diversity size was higher in adult than young animals independently of sex and season of sampling (Fig. 4B).

## Discussion

The helminth fauna of warthogs at HNR and KNP was moderately species rich and coincided largely with that of warthogs examined in the Pongola Game Reserve (PGR) in KwaZulu-Natal Province, South Africa (Junker *et al*., 2019), at various localities in Limpopo Province (van Wyk and Boomker, 2011) and Namibia (Horak *et al*., 1983). Gastrointestinal helminth species that have consistently been found in warthogs at several localities in southern Africa and form the core of parasite assemblages of this host are the nematodes *A. phacochoeri*, *D. mocambiquei*, *D. mwanzae*, *I. tuberculata*, *M. hamata*, *M. pugnicaudata*, *P. sexalatus* and *Probstmayria* sp. as well as cestodes of the genus *Moniezia* (Horak *et al*., 1983; van Wyk and Boomker, 2011; Junker *et al*., 2019). The gap between helminth species present in infracommunities at a given locality and those that are known parasites of warthogs, i.e. dark diversity, is likely influenced by local environmental conditions that enable the survival of free-living stages and intermediate hosts (Junker *et al*., 2019). Furthermore, varying sets of sympatric hosts might add to the available pool of helminth species at a given locality (Huang *et al*., 2014; Junker *et al*., 2022). It is noteworthy, that warthog–helminth networks at HNR and KNP were more species rich than those at other localities. In addition, at HNR, dark diversity was higher in adult than young warthogs, possibly because of a better immune response to parasite infection in adults (Fellis *et al*., 2003; Tinsley *et al*., 2012; Nielsen *et al*., 2020). In support of this, the prevalence of *Moniezia* sp. at HNR was 76.2% in young warthogs, but dropped to 5% in adults, which typically acquire immunity after the first infection (Reinecke, 1983).

### Nestedness

Warthog-parasite networks at both localities followed a nested pattern, although less pronounced in KNP than HNR. Our analyses demonstrated the influence of both host sex and age, as well as season of sampling on the structure and dynamics within these networks. Interestingly, and as shown in previous studies (Zelmer *et al*., 2004; Chaisiri *et al*., 2017; Spickett *et al*., 2019; Junker *et al*., 2022), the effect of a given factor on the same metric can vary between localities.

At HNR, nestedness rank was significantly higher in adult than young warthogs in the wet season. A number of studies have shown parasite infracommunities of adult hosts to be more species rich when compared to young animals. They associated this with an increase in age and size leading to higher mobility and/or food intake, which in turn increases exposure to free-living infective stages or intermediate hosts of parasites (Guégan and Hugueny, 1994; Rohde *et al*., 1998; Zelmer *et al*., 2004). Furthermore, females of ruminants have been shown to be more susceptible to parasite infection during the periparturient and lactating period due to a loss of immunity (Barger, 1993; Beasley *et al*., 2010). In warthogs, this period extends from November to February (Somers *et al*., 1995), and could thus add to the above phenomenon. On the other hand, adult male warthogs spend much time defending females rather than feeding during the mating season from May to June (Somers *et al*., 1995), which coincides with the dry season (May to August).

Male warthogs contributed more to nestedness than females at HNR. Warthog males are larger than females, offering larger niche space for parasites, have larger home ranges, and disperse more than females, including roaming between females during mating season (Somers *et al*., 1994). Consequently, males have an increased chance of acquiring diverse parasite infracommunities, which contributes to nestedness (Zelmer *et al*., 2004; González and Poulin, 2005; Cardoso *et al*., 2021).

In the KNP, both host sex and season influenced nestedness contribution. In the wet season, the pattern was similar to that seen at HNR, but was converse in the dry season, when values for nestedness contribution were higher in females than males. As mentioned above, adult male warthogs foregoing foraging in the interest of defending a possible mate might reduce exposure to parasites.

Values of nestedness resultant where higher in females than males in KNP during the dry season, but higher in males than females during the wet season. This follows the same pattern as nestedness contribution and is likely due to the same physiological and behavioural gender differences as highlighted above. Environmental gradients between the 2 localities might mediate the extent to which host heterogeneity influences parasite transmission in relation to host sex and age classes of warthogs (Spickett *et al*., 2019; Junker *et al*., 2022).

### Host specialization and centrality

At HNR, values of individual host specialization (d’) were higher in the dry than the wet season; indicating that the distribution of helminth species in the dry season was patchier than in the wet season. The observed differences likely reflect varying tolerance limits of different helminth species towards environmental stressors such as temperature and moisture (Rossanigo and Gruner, 1995; O'Connor *et al*., 2006; Costa-Neto *et al*., 2019), allowing only those with wider tolerance ranges to persist during the dry season. The reduced risk of desiccation during the wet season especially profits helminth species with direct life cycles whose infective stages are exposed to the external environment and which comprise the majority of species in infracommunities in warthogs.

At KNP, on the other hand, d’ values were significantly higher in young than adult warthogs, suggesting that helminth species with poor colonization ability might depend on young animals with naïve immune systems.

Interaction between age and season led to significantly higher values of centrality in young when compared to adult warthogs at HNR in the wet season. A converse effect of age on helminth–parasite networks was found in nyalas in Mkhuze, where values of centrality were higher in adult than young females, and in iMfolozi, values of centrality were higher in male than female nyalas irrespective of the age (Junker *et al*., 2022). Such network variation is likely caused by differences in host mobility, foraging preferences, immunity or sociality between the sexes or associated with an increase in age and influenced by habitat heterogeneity and climate conditions at a given locality (Fellis *et al*., 2003; Zelmer *et al*., 2004; Foata *et al*., 2006; Negovetich *et al*., 2006; Saeed *et al*., 2006; Bellay *et al*., 2020).

Young warthogs at HNR contributed more to parasite transmission in the network than adults. Piglets form part of matriarchal groups and subsequently reorganise into yearling groups from which younger males move on to bachelor groups. In contrast, adult males are typically solitary and females, while organized in matriarchal groups during much of the year, leave the group during the farrowing season (Somers *et al*., 1995). High host population density is known to increase the probability of contact between the infective stages of a parasite and its host (Arneberg *et al*., 1998; Arneberg, 2002; Cardoso *et al*., 2021). Their higher sociality, combined with changing group associations and related habitat shifts, likely expose young warthogs to a larger variety of helminth taxa when compared to adults.

### Beta diversity

Irrespective of their age or sex, warthogs sampled during the dry season at HNR contributed more to beta diversity of infracommunities than those sampled during the wet season. A reduced and uneven spread of infective stages in the environment during the dry season, resulting from increased selective pressure of adverse climatic conditions (Brooker *et al*., 2006), might explain this.

In general, the effects of sex, age and season were more pronounced in HNR than in KNP, but they also often varied at the 2 localities. While all sampling sites of warthogs were located in the Granite Lowveld, local habitat differences associated with variations in the potential intermediate and final host fauna of parasites would have been more pronounced between sampling sites in HNR and KNP than within HNR and KNP themselves and would have affected transmission dynamics at the 2 localities.

In conclusion, the present study suggests that helminth communities of warthogs are structured networks that are influenced by numerous internal factors, such as host sex and age as well as the dietary and/or behavioural patterns associated with these. Furthermore, external factors, such as climate, habitat diversity and co-occurring host species at a given locality contribute to network organization. Varying combinations of these can result in a multitude of responses shaping each host–parasite network individually. It is therefore only with great circumspection that network patterns observed at 1 locality should be applied to the same host–parasite system in a different geographic setting.

## Data Availability

All data generated or analysed during this study are included in this published article. The datasets used and/or analysed are available from the corresponding author upon reasonable request.

## Supplementary material

For supplementary material accompanying this paper visit https://doi.org/10.1017/S0031182023000719. click here to view supplementary material

## References

[ref1] Almeida-Neto M and Ulrich W (2011) A straightforward computational approach for measuring nestedness using quantitative matrices. Environmental Modelling & Software 26, 173–178.

[ref2] Almeida-Neto M, Guimarães P, Guimarães PR Jr, Loyola RD and Ulrich W (2008) A consistent metric for nestedness analysis in ecological systems: reconciling concept and measurement. Oikos 117, 1227–1239.

[ref3] Amundson CL, Traub NJ, Smith-Herron AJ and Flint PL (2016) Helminth community structure in two species of Arctic-breeding waterfowl. International Journal of Parasitology: Parasites and Wildlife 5, 263–272. 10.1016/j.ijppaw.2016.09.002 PMC5040642 27709067

[ref4] Arita HT (2016) Species co-occurrence analysis: pairwise versus matrix-level approaches. Global Ecology and Biogeography 25, 1397–1400.

[ref5] Arneberg P (2002) Host population density and body mass as determinants of species richness in parasite communities: comparative analyses of directly transmitted nematodes of mammals. Ecography 25, 88–94.

[ref6] Arneberg P, Skorping A, Grenfell B and Read AF (1998) Host densities as determinants of abundance in parasite communities. Proceedings of the Royal Society of London. Series B: Biological Sciences 265, 1283–1289.

[ref7] Atmar W and Patterson BD (1993) The measure of order and disorder in the distribution of species in fragmented habitat. Oecologia 96, 373–382. 28313653 10.1007/BF00317508

[ref8] Barger IA (1993) Influence of sex and reproductive status on susceptibility of ruminants to nematode parasitism. International Journal for Parasitology 23, 463–469. 8354597 10.1016/0020-7519(93)90034-v

[ref9] Barton K (2020) MuMIn: Multi-Model Inference. R package version 1.43.17. Available at https://CRAN.R-project.org/package=MuMIn.

[ref10] Bascompte J, Jordano P, Melian CJ and Olesen JM (2003) The nested assembly of plant–animal mutualistic networks. Proceedings of the National Academy of Sciences USA 100, 9383–9387. 10.1073/pnas.1633576100 PMC170927 12881488

[ref11] Baselga A (2010) Partitioning the turnover and nestedness components of beta diversity. Global Ecology and Biogeography 19, 134–143.

[ref12] Baselga A, Orme D, Villeger S, De Bortoli J, Leprieur F, Logez M, Martinez-Santalla S, Martin-Devasa R, Gomez-Rodriguez C and Crujeiras R (2023) betapart: Partitioning Beta Diversity into Turnover and Nestedness Components. R package version 1.6. Available at https://CRAN.R-project.org/package=betapart

[ref13] Beasley AM, Kahn LP and Windon RG (2010) The periparturient relaxation of immunity in Merino ewes infected with *Trichostrongylus colubriformis*: endocrine and body compositional responses. Veterinary Parasitology 168, 51–59. 20092949 10.1016/j.vetpar.2009.12.012

[ref14] Belem AMG (2012) Gastro-intestinal parasites of warthogs (*Phacochoerus africanus*) from the Nazinga Game Ranch of Burkina Faso. Animal Health and Production 60, 199–204.

[ref15] Bellay S, Oda FH, Almeida-Neto M, de Oliveira EF, Takemoto RM and Balbuena JA (2020) Host age predicts parasite occurrence, richness, and nested infracommunities in a pilot whale–helminth network. Parasitology Research 119, 2237–2244. 32451718 10.1007/s00436-020-06716-1

[ref16] Beveridge I (2014) A review of the genus *Paramoniezia* Maplestone et Southwell, 1923 (Cestoda: Anoplocephalidae), with a new genus, *Phascolocestus*, from wombats (Marsupialia) and redescriptions of *Moniezia mettami* Baylis, 1934 and *Moniezia phacochoeri* (Baylis, 1927) comb. n. from African warthogs (Artiodactyla). Folia Parasitologica 61, 21. 24684050

[ref17] Blüthgen N (2010) Why network analysis is often disconnected from community ecology: a critique and an ecologist's guide. Basic Applied Ecology 11, 185–195.

[ref18] Blüthgen N, Menzel F and Blüthgen N (2006) Measuring specialization in species interaction networks. BMC Ecology 6, 1–12. 16907983 10.1186/1472-6785-6-9 PMC1570337

[ref19] Boomker J, Horak IG, Booyse DG and Meyer S (1991) Parasites of warthogs, *Phacochoerus aethiopicus*, in the eastern Transvaal. Onderstepoort Journal of Veterinary Research 58, 195–202. 1923382

[ref20] Brooker S, Clements AC and Bundy DA (2006) Global epidemiology, ecology and control of soil-transmitted helminth infections. Advances in Parasitology 62, 221–261. 16647972 10.1016/S0065-308X(05)62007-6 PMC1976253

[ref21] Calvete C, Estrada R, Lucientes J, Estrada A and Telletxea I (2003) Correlates of helminth community in the red-legged partridge (*Alectoris rufa* L.) in Spain. Journal of Parasitology 89, 445–451. 12880240 10.1645/0022-3395(2003)089[0445:COHCIT]2.0.CO;2

[ref22] Cardoso TS, Andreazzi CS, Maldonado Junior A and Gentile R (2021) Functional traits shape small mammal-helminth network: patterns and processes in species interactions. Parasitology 148, 947–955. 33879271 10.1017/S0031182021000640 PMC8193565

[ref23] Carmona CP and Pärtel M (2021) Estimating probabilistic site-specific species pools and dark diversity from co-occurrence data. Global Ecology and Biogeography 30, 316–326.

[ref24] Carney JP and Dick TA (2000) Helminth communities of yellow perch (*Perca flavescens* (Mitchill)): determinants of pattern. Canadian Journal of Zoology 78, 538–555.

[ref25] Chaisiri K, Chou M, Siew CC, Morand S and Ribas A (2017) Gastrointestinal helminth fauna of rodents from Cambodia: emphasizing the community ecology of host–parasite associations. Journal of Helminthology 91, 726–738. 27905270 10.1017/S0022149X16000869

[ref26] Chen C, Zhan C and Wang Y (2022) Do functional and phylogenetic nestedness follow the same mechanisms as taxonomic nestedness? Evidence from amphibians in the largest archipelago of China. Journal of Animal Ecology 91, 2424–2436. 36260356 10.1111/1365-2656.13824

[ref27] Corn JL, Pence DB and Warren RJ (1985) Factors affecting the helminth community structure of adult collared peccaries in southern Texas. Journal of Wildlife Diseases 21, 254–263. 4032623 10.7589/0090-3558-21.3.254

[ref28] Costa-Neto SF, Cardoso TS, Boullosa RG, Maldonado A and Gentile R (2019) Metacommunity structure of the helminths of the black-eared opossum *Didelphis aurita* in peri-urban, sylvatic and rural environments in south-eastern Brazil. Journal of Helminthology 93, 720–731. 30220264 10.1017/S0022149X18000780

[ref29] Cribari-Neto F and Zeileis A (2010) Beta regression in R. Journal of Statistical Software 34, 1–24.

[ref30] Csardi G and Nepusz T (2006) The igraph software package for complex network research. International Journal of Computer Systems, 1695, 1–9. Available at http://igraph.org

[ref31] de-la-Muela N, Hernández-de-Luján S and Ferre I (2001) Helminths of wild boar in Spain. Journal of Wildlife Diseases 37, 840–843. 11763752 10.7589/0090-3558-37.4.840

[ref32] Dormann CF (2011) How to be a specialist? Quantifying specialisation in pollination networks. Network Biology 1, 1–20.

[ref33] Dormann CF, Gruber B and Fruend J (2008) Introducing the bipartite package: analysing ecological networks. R News 8, 8–11.

[ref34] Dray S, Bauman D, Blanchet G, Borcard D, Clappe S, Guenard G, Jombart T, Larocque G, Legendre P, Madi N and Wagner HH (2018) adespatial: Multivariate Multiscale Spatial Analysis. R package version 0.2–0. Available at https://CRAN.R-project.org/package=adespatial

[ref35] Fellis KJ, Negovetich NJ, Esch GW, Horak IG and Boomker J (2003) Patterns of association, nestedness, and species co-occurrence of helminth parasites in the greater kudu, *Tragelaphus strepsiceros*, in the Kruger National Park, South Africa, and the Etosha National Park, Namibia. Journal of Parasitology 89, 899–907. 14627135 10.1645/GE-3189

[ref36] Foata J, Mouillot D, Culioli JL and Marchand B (2006) Influence of season and host age on wild boar parasites in Corsica using indicator species analysis. Journal of Helminthology 80, 41–45. 16469171 10.1079/joh2005329

[ref37] González MT and Poulin R (2005) Nested patterns in parasite component communities of a marine fish along its latitudinal range on the Pacific coast of South America. Parasitology 131, 569–577. 16174422 10.1017/S0031182005007900

[ref38] Gotelli NJ (2000) Null model analysis of species cooccurrence patterns. Ecology 81, 2606–2621.

[ref39] Guégan JF and Hugueny B (1994) A nested parasite species subset pattern in tropical fish: host as a major determinant of parasite infracommunity structure. Oecologia 100, 184–189. 28307042 10.1007/BF00317145

[ref40] Horak IG, Biggs HC, Hanssen TS and Hanssen RE (1983) The prevalence of helminth and arthropod parasites of warthog, *Phacochoerus aethiopicus*, in South West Africa/Namibia. Onderstepoort Journal of Veterinary Research 50, 145–148. 6634088

[ref41] Horak IG, Boomker J and Potgieter FT (1988) Parasites of domestic and wild animals in South Africa. XXIII. Helminth and arthropod parasites of warthogs, *Phacochoerus aethiopicus*, in the eastern Transvaal Lowveld. Onderstepoort Journal of Veterinary Research 55, 145–152. 3194114

[ref42] Huang S, Bininda Emonds OR, Stephens PR, Gittleman JL and Altizer S (2014) Phylogenetically related and ecologically similar carnivores harbour similar parasite assemblages. Journal of Animal Ecology 83, 671–680. 24289314 10.1111/1365-2656.12160

[ref43] Isomursu M, Rätti O, Helle P and Hollmén T (2006) Sex and age influence intestinal parasite burden in three boreal grouse species. Journal of Avian Biology 37, 516–522.

[ref44] Junker K, Spickett A, Swanepoel M, Krasnov BR, Boomker J and Hoffman LC (2019) Gastrointestinal helminths from the common warthog, *Phacochoerus africanus* (Gmelin)(Suidae), in KwaZulu-Natal Province, South Africa, with comments on helminths of Suidae and Tayassuidae worldwide. Parasitology 146, 1541–1549. 31106726 10.1017/S0031182019000684

[ref45] Junker K, Boomker J, Horak IG and Krasnov BR (2022) Impact of host sex and age on the diversity of endoparasites and structure of individual-based host–parasite networks in nyalas (*Tragelaphus angasii* Angas) from three game reserves in KwaZulu-Natal province, South Africa. Parasitology Research 121, 3249–3267. 36071296 10.1007/s00436-022-07653-x

[ref46] Krasnov BR, Stanko M and Morand S (2006) Are ectoparasite communities structured? Species co-occurrence, temporal variation and null models. Journal of Animal Ecology 75, 1330–1339. 17032365 10.1111/j.1365-2656.2006.01156.x

[ref47] Krasnov BR, Stanko M, Khokhlova IS, Shenbrot GI, Morand S, Korallo-Vinarskaya NP and Vinarski MV (2010) Nestedness and *β*-diversity in ectoparasite assemblages of small mammalian hosts: effects of parasite affinity, host biology and scale. Oikos 120, 630–639.

[ref48] Krasnov BR, Shenbrot GI, Warburton EM, Van Der Mescht L, Surkova EN, Medvedev SG, Pechnikova N, Ermolova N, Kotti BK and Khokhlova IS (2019) Species and site contributions to *β*-diversity in fleas parasitic on the Palearctic small mammals: ecology, geography and host species composition matter the most. Parasitology 146, 653–661. 30430954 10.1017/S0031182018001944

[ref49] Legendre P and De Cáceres M (2013) Beta diversity as the variance of community data: dissimilarity coefficients and partitioning. Ecology Letters 16, 951–963. 23809147 10.1111/ele.12141

[ref50] Lewis RJ, Szava-Kovats R and Pärtel M (2016) Estimating dark diversity and species pools: an empirical assessment of two methods. Methods in Ecology and Evolution 7, 104–113.

[ref51] Martins PM, Poulin R and Gonçalves-Souza T (2021) Drivers of parasite *β*-diversity among anuran hosts depend on scale, realm and parasite group. Philosophical Transactions of the Royal Society B 376, 20200367. 10.1098/rstb.2020.0367 PMC8450629 34538138

[ref52] Martínez-Guijosa J, Martínez-Carrasco C, López-Olvera JR, Fernández-Aguilar X, Colom-Cadena A, Cabezón O and Serrano E (2015) Male-biased gastrointestinal parasitism in a nearly monomorphic mountain ungulate. Parasites and Vectors 8, 1–5. 25888900 10.1186/s13071-015-0774-9 PMC4408582

[ref53] Morand S, McIntyre M and Baylis M (2014) Domesticated animals and human infectious diseases of zoonotic origins: domestication time matters. Infection, Genetics and Evolution 24, 76–81. 10.1016/j.meegid.2014.02.013 24642136

[ref54] Mucina L and Rutherford MC (2006) The Vegetation of South Africa, Lesotho and Swaziland. Strelitzia 19. Pretoria, South Africa: South African National Biodiversity Institute.

[ref55] Negovetich NJ, Fellis KJ, Esch GW, Horak IG and Boomker J (2006) An examination of the infracommunities and component communities from impala (*Aepyceros melampus*) in the Kruger National Park, South Africa. Journal of Parasitology 92, 1180–1190. 17304792 10.1645/GE-934R.1

[ref56] Nielsen ÓK, Morrill A, Skírnisson K, Stenkewitz U, Pálsdóttir GR and Forbes MR (2020) Host sex and age typically explain variation in parasitism of rock ptarmigan: implications for identifying determinants of exposure and susceptibility. Journal of Avian Biology 51, e02472.

[ref57] Norton J, Lewis JW and Rollinson D (2004) Temporal and spatial patterns of nestedness in eel macroparasite communities. Parasitology 129, 203–211. 15376779 10.1017/s0031182004005517

[ref58] O'Connor LJ, Walkden-Brown SW and Kahn LP (2006) Ecology of the free-living stages of major trichostrongylid parasites of sheep. Veterinary Parasitology 142, 1–15. 17011129 10.1016/j.vetpar.2006.08.035

[ref59] Opsahl T (2009) Structure and Evolution of Weighted Networks. London, UK: University of London (Queen Mary College), pp. 104–122. Available at http://toreopsahl.com/publications/thesis/; http://toreopsahl.com/tnet/

[ref60] Pärtel M, Szava-Kovats R and Zobel M (2011) Dark diversity: shedding light on absent species. Trends in Ecology & Evolution 26, 124–128. 21195505 10.1016/j.tree.2010.12.004

[ref61] Pärtel M, Szava-Kovats R and Zobel M (2013) Community completeness: linking local and dark diversity within the species pool concept. Folia Geobotanica 48, 307–317.

[ref62] Patterson BD and Atmar W (1986) Nested subsets and the structure of insular mammalian faunas and archipelagos. Biological Journal of the Linnean Society 28, 65–82.

[ref63] Pilosof S, Morand S, Krasnov BR and Nunn CL (2015) Potential parasite transmission in multi-host networks based on parasite sharing. PLoS One 10, 1–19. doi: 10.1371/journal.pone.0117909 PMC4352066 25748947

[ref64] Poulin R and Valtonen ET (2001) Nested assemblages resulting from host size variation: the case of the endoparasite communities in fish hosts. International Journal for Parasitology 31, 1194–1204. 11513888 10.1016/s0020-7519(01)00262-4

[ref65] R Core Team (2021) R: A Language and Environment for Statistical Computing. Vienna, Austria: R Foundation for Statistical Computing, URL https://www.R-project.org/

[ref66] Reinecke RK (1983) Veterinary Helminthology. Durban, South Africa: Butterworth Publishers.

[ref67] Rohde K, Worthen WB, Heap M, Hugueny B and Guégan JF (1998) Nestedness in assemblages of metazoan ecto-and endoparasites of marine fish. International Journal for Parasitology 28, 543–549. 9602374 10.1016/s0020-7519(98)00013-7

[ref68] Ronk A, Szava-Kovats R and Pärtel M (2015) Applying the dark diversity concept to plants at the European scale. Ecography 38, 1015–1025.

[ref69] Rossanigo CE and Gruner L (1995) Moisture and temperature requirements in faeces for the development of free-living stages of gastrointestinal nematodes of sheep, cattle and deer. Journal of Helminthology 69, 357–362. 8583130 10.1017/s0022149x00014954

[ref70] Rynkiewicz EC, Fenton A and Pedersen AB (2019) Linking community assembly and structure across scales in a wil mouse parasite community. Ecology and Evolution 9, 13752–13763. 31938479 10.1002/ece3.5785 PMC6953566

[ref71] Saeed I, Maddox-Hyttel C, Monrad J and Kapel CMO (2006) Helminths of red foxes (*Vulpes vulpes*) in Denmark. Veterinary Parasitology 139, 168–179. 16580775 10.1016/j.vetpar.2006.02.015

[ref72] Skinner JD and Chimimba CT (2005) The Mammals of the Southern African Subregion, 3rd Edn. Cape Town, South Africa: Cambridge University Press.

[ref73] Somers MJ, Penzhorn BL and Rasa AE (1994) Home range size, range use and dispersal of warthogs in the eastern Cape, South Africa. Journal of African Zoology 108, 361–374.

[ref74] Somers MJ, Rasa OAE and Penzhorn BL (1995) Group structure and social behaviour of warthogs *Phacochoerus aethiopicus*. Acta Theriologica 40, 257–282.

[ref75] Spickett A, Junker K, Krasnov BR, Haukisalmi V and Matthee S (2017) Helminth parasitism in two closely related South African rodents: abundance, prevalence, species richness and impinging factors. Parasitology Research 116, 1395–1409. 28281100 10.1007/s00436-017-5419-9

[ref76] Spickett A, van der Mescht L, Junker K, Krasnov BR, Haukisalmi V and Matthee S (2019) Beta diversity of gastrointestinal helminths in two closely related South African rodents: species and site contributions. Parasitology Research 118, 2863–2875. 31399870 10.1007/s00436-019-06411-w

[ref77] Tinsley R, Stott L, York J, Everard A, Chapple S, Jackson J, Viney M and Tinsley MC (2012) Acquired immunity protects against helminth infection in a natural host population: long-term field and laboratory evidence. International Journal for Parasitology 42, 931–938. 22906507 10.1016/j.ijpara.2012.07.006

[ref78] Troncy PM, Graber M and Thal J (1972) *Probstmayria suis* n. sp. (Nematode, Atractidae), parasite de Suidae. Bulletin du Muséum National d'Histoire Naturelle( 3e série, no. 94, Zoologie) 73, 1313–1316.

[ref79] Ulrich W, Almeida-Neto M and Gotelli NJ (2009) A consumer's guide to nestedness analysis. Oikos 118, 3–17.

[ref80] van Wyk IC and Boomker J (2011) Parasites of South African wildlife: XIX. The prevalence of helminths in some common antelopes, warthogs and a bushpig in the Limpopo province, South Africa. Onderstepoort Journal of Veterinary Research 78, 1–11. 10.4102/ojvr.v78i1.308 23327219

[ref81] Wang Y, Ding P, Chen S and Zheng G (2013) Nestedness of bird assemblages on urban woodlots: implications for conservation. Landscape and Urban Planning 111, 59–67.

[ref82] Warburton EM, van der Mescht L, Khokhlova IS, Krasnov BR and Vonhof MJ (2018) Nestedness in assemblages of helminth parasites of bats: a function of geography, environment, or host nestedness? Parasitology Research 117, 1621–1630. 29594347 10.1007/s00436-018-5844-4

[ref83] Zelmer DA, Paredes-Calderón L, León-Règagnon V and García-Prieto L (2004) Nestedness in colonization-dominated systems: helminth infracommunities of *Rana vaillanti* Brocchi (Anura: Ranidae) in Los Tuxtlas, Veracruz, Mexico. Journal of Parasitology 90, 705–710. 15357057 10.1645/GE-3316

