# Supplementary material for: Nestedness and beta diversity of gastrointestinal helminth communities in common warthogs, Phacochoerus africanus (Suidae), at 2 localities in South Africa
Source: Parasitology. 2023 Aug 9;150(10):911–21. doi: 10.1017/S0031182023000719 (PMC10577656; doi:10.1017/S0031182023000719)
Supplement: Supplementary file 1 [file S0031182023000719sup.zip › S0031182023000719sup001.docx]

**Supplementary material. Table S1.** Prevalence and abundance (range; total count) of parasites collected from common warthogs, *Phacochoerus* *africanus* (Gmelin), in the Kruger National Park and the Hoedspruit Nature Reserve, South Africa.

| **Locality** | **Kruger National Park (n = 54)** | | | **Hoedspruit Nature Reserve (n = 41)** | | |
| --- | --- | --- | --- | --- | --- | --- |
| **Parasite taxon** | ***N*** | **Prev. (%)** | **Range; total count** | ***N*** | **Prev. (%)** | **Range; total count** |
| **Nematoda** |  |  |  |  |  |  |
| *Ascaris phacochoeri* Gedoelst, 1916 | 16 | 29.6 | 0–7; 35 | 18 | 43.9 | 0–54; 146 |
| *Ascaris pahcochoeri* L4 | 1 | 1.9 | 0–27; 27 | - | - | - |
| *Cooperia hungi* Mönnig, 1932 | - | - | - | 1 | 2.4 | 0–100; 100 |
| *Daubneyia mocambiquei* Ortlepp, 1964 | 45 | 83.3 | 0–5760; 52605 | 39 | 95.1 | 0–30700; 98565 |
| *Daubneyia mwanzae* (Daubney, 1924) | 26 | 48.1 | 0–1440; 5185 | 40 | 97.6 | 0–6220; 41774 |
| *Daubneyia* sp. L4 | 17 | 31.5 | 0–500; 2256 | 16 | 39.0 | 0–916; 2995 |
| *Impalaia tuberculata* Mönnig, 1923 | 6 | 11.1 | 0–189; 566 | 5 | 12.2 | 0–70; 150 |
| *Impalaia tuberculata* L4 | 7 | 13.0 | 0–113; 236 | nd | nd | nd |
| *Murshidia hamata* Daubney, 1923 | 41 | 75.9 | 0–38200; 87680 | 41 | 100 | 25–5860; 56095 |
| *Murshidia pugnicaudata* (Leiper, 1909) | 36 | 66.7 | 0–8600; 18718 | 40 | 97.6 | 0–1130; 10803 |
| *Murshidia* sp. L4 | 17 | 31.5 | 0–2320; 4411 | 13 | 31.7 | 0–120; 340 |
| *Physocephalus sexalatus* (Molin, 1860) | 20 | 37.0 | 0–180; 666 | 33 | 80.5 | 0–3550; 10104 |
| *Physocephalus* sp. L4 | 8 | 14.8 | 0–100; 232 | 3 | 7.3 | 0–40; 80 |
| *Probstmayria* sp. | 54 | 100 | Millions | 41 | 100 | Millions |
| *Strongyloides* sp. | 3 | 5.6 | 0–75; 116 | - | - | - |
| *Streptopharagus* sp. | 2 | 3.7 | 0–1; 2 | - | - | - |
| *Trichostrongylus deflexus* Boomker & Reinecke, 1989 | 4 | 7.4 | 0–538; 743 | 2 | 4.9 | 0–10; 20 |
| *Trichostrongylus falculatus* Ransom, 1912 | 1 | 1.9 | 0–50; 50 | - | - | - |
| *Trichostrongylus thomasi* Mönnig, 1932 | 8 | 14.8 | 0–50; 139 | 4 | 9.8 | 0–30; 60 |
| *Trichostrongylus* sp.^a^ | 1 | 1.9 | 0–75; 75 | 6 | 14.6 | 0–10; 60 |
| *Trichostrongylus* sp. L4 | 41 | 75.9 | 0–2820; 7982 | nd | nd | nd |
| *Trichuris* sp. | 1 | 1.9 | 0–10; 10 | - | - | - |
| **Cestoda** |  |  |  |  |  |  |
| *Moniezia* sp. | 11 | 20.4 | 0–20; 58 | 17 | 41.5 | 0–25; 162 |
| **Trematoda** |  |  |  |  |  |  |
| *Schistosoma* sp. | 1 | 1.9 | 0–15; 15 | - | - | - |

^a^Only females present in a given host; L4 – fourth-stage larvae; Prev. – prevalence; nd – not done (see text)
