# Supplementary material for: Nestedness and beta diversity of gastrointestinal helminth communities in common warthogs, Phacochoerus africanus (Suidae), at 2 localities in South Africa
Source: Parasitology. 2023 Aug 9;150(10):911–21. doi: 10.1017/S0031182023000719 (PMC10577656; doi:10.1017/S0031182023000719)
Supplement: Supplementary file 1 [file S0031182023000719sup.zip › S0031182023000719sup002.docx]

**Supplementary material. Table S2** Classification and life history traits of helminths of warthogs, *Phacochoerus* *africanus* (Gmelin), collected in the Kruger National Park and Hoedspruit Nature Reserve, South Africa.

| **Parasite taxon** | **Family** | **Site in host** | **Life cycle** | **Transmission mode** |
| --- | --- | --- | --- | --- |
| **Phylum: Nematoda** |  |  |  |  |
| **Class: Chromadorea** |  |  |  |  |
| **Order: Strongylida** |  |  |  |  |
| *Daubneyia mocambiquei* Ortlepp, 1964 | Chabertiidae | Large intestine | Direct | Accidental ingestion of L3 |
| *Daubneyia mwanzae* (Daubney, 1924) | Chabertiidae | Large intestine | Direct | Accidental ingestion of L3 |
| *Murshidia hamata* Daubney, 1923 | Strongylidae | Large intestine | Direct | Accidental ingestion of L3 |
| *Murshidia pugnicaudata* (Leiper, 1909) | Strongylidae | Large intestine | Direct | Accidental ingestion of L3 |
| *Cooperia hungi* Mönnig, 1932 | Cooperiidae | Small intestine | Direct | Accidental ingestion of L3 |
| *Impalaia tuberculata* Mönnig, 1923 | Cooperiidae | Small intestine | Direct | Accidental ingestion of L3 |
| *Trichostrongylus deflexus* Boomker & Reinecke, 1989 | Trichostrongylidae | Small intestine | Direct | Accidental ingestion of L3 |
| *Trichostrongylus falculatus* Ransom, 1912 | Trichostrongylidae | Small intestine | Direct | Accidental ingestion of L3 |
| *Trichostrongylus thomasi* Mönnig, 1932 | Trichostrongylidae | Small intestine | Direct | Accidental ingestion of L3 |
| *Trichostrongylus* sp.^a^ | Trichostrongylidae | Small intestine | Direct | Accidental ingestion of L3 |
| **Order: Ascaridida** |  |  |  |  |
| *Ascaris phacochoeri* Gedoelst, 1916 | Ascarididae | Small intestine | Direct | Accidental ingestion of eggs |
| *Probstmayria* sp. | Atractidae | Large intestine | Direct | Accidental ingestion of various larval stages; **autoinfection** |
| **Order: Rhabditida** |  |  |  |  |
| *Strongyloides* sp. | Strongyloididae | Small intestine | Direct | Percutaneous invasion |
| **Order: Spirurida** |  |  |  |  |
| *Physocephalus sexalatus* (Molin, 1860) | Spirocercidae | Stomach | Indirect | Accidental ingestion of intermediate host |
| *Streptopharagus* sp. | Spirocercidae | Stomach | Indirect | Accidental ingestion of intermediate host |
| **Class: Enoplea** |  |  |  |  |
| **Order: Trichinellida** |  |  |  |  |
| *Trichuris* sp. | Trichuridae | Large intestine | Direct | Accidental ingestion of eggs |
| **Phylum: Platyhelminthes** |  |  |  |  |
| **Class: Cestoda** |  |  |  |  |
| **Order: Cyclocephallidea** |  |  |  |  |
| *Moniezia* sp. | Anoplocephalidae | Small intestine | Indirect | Accidental ingestion of intermediate host |
| **Class: Trematoda** |  |  |  |  |
| **Order: Diplostomida** |  |  |  |  |
| *Schistosoma* sp. | Schistosomatidae | Mesenteric veins | Indirect | Percutaneous invasion of both intermediate and definitive host |

^a^Only females present in a given host; L3 – Infective larva
